# Supplementary material for: Emergence of NDM-1-Producing Pseudomonas aeruginosa Nosocomial Isolates in Attica Region of Greece
Source: Microorganisms. 2024 Aug 23;12(9):1753. doi: 10.3390/microorganisms12091753 (PMC11434298; doi:10.3390/microorganisms12091753)
Supplement: Supplementary file 1 [file microorganisms-12-01753-s001.zip › microorganisms-3163709-supplementary.pdf]

microorganisms MDPI

Supplementary Table S1. Antimicrobial susceptibility profiles of NDM-1-producing *P. aeruginosa* isolates.

	MIC [mg/L]									
	VITEK2 system									Broth microdilution
Isolate no.	PIP-TAZ	CAZ	FEP	ATM	IPM	MEM	AMK	GEN	CIP	COL
**9953**	≥128	≥64	≥64	16	≥16	≥16	≥64	≥16	≥4	2
**9959**	≥128	≥64	≥32	16	≥16	≥16	≥64	≥16	≥4	1
**9954**	≥128	≥64	≥32	16	≥16	≥16	≥64	≥16	≥4	1
**9955**	≥128	≥64	≥64	16	≥16	≥16	≥64	≥16	≥4	0.5
**9956**	≥128	≥64	≥32	16	≥16	≥16	≥64	≥16	≥4	0.5
**9957**	≥128	≥64	≥32	16	≥16	≥16	≥64	≥16	≥4	0.5
**9958**	≥128	≥64	≥32	16	≥16	≥16	≥64	≥16	≥4	0.5
**9951**	≥128	≥64	≥32	16	≥16	≥16	≥64	≥16	≥4	1
**9960**	≥128	≥64	≥32	16	≥16	≥16	≥64	≥16	≥4	0.5
**9962**	≥128	≥64	≥32	16	≥16	≥16	≥64	≥16	≥4	1
**9963**	≥128	≥64	≥32	16	≥16	≥16	≥64	≥16	≥4	0.5
**9964**	≥128	≥64	≥32	16	≥16	≥16	≥64	≥16	≥4	0.5
**9965**	≥128	≥64	≥32	≥64	≥16	≥16	≥64	≥16	≥4	1
**9966**	≥128	≥64	≥32	16	≥16	≥16	≥64	≥16	≥4	0.5
**9952**	≥128	≥64	≥64	16	≥16	≥16	≥64	≥16	≥4	0.5
**9912**	>64	>32	>32	16	>8	>8	>32	>8	>2	2
**10071**	>64	>32	>32	16	>8	>8	>32	>8	>2	2

Supplementary Table S2. MLST, DLST types and antibiotic resistance genetic determinants detected in the study isolates.

				MDR-flow-chip analysis	WGS analysis	
**a/a**	**Isolate no**	**MLST**	**DLST**	**ARGs and AMR chromosomal point mutations**	**AMRFinder_ARGs**	**AMR chromosomal point mutations**
**1**	**9953**	773	26 46	NDM, sul-1, rmtB, mut gyrA_T83I	aadA11, aph[3']-IIb, blaNDM-1, blaOXA-395, blaPDC-16, catB7, crpP, fosA, qacEdelta1, qnrVC1, rmtB4, sul1, tet[G], floR2	parC_S87L gyrA_T83I
**2**	**9959**	773		NDM, sul-1, rmtB, mut. gyrA_T83I		
**3**	**9954**	773		NDM, sul-1, rmtB, mut gyrA_T83I		
**4**	**9955**	773		NDM, sul-1, rmtB, mut gyrA_T83I		
**5**	**9956**	773		NDM, sul-1, rmtB, mut gyrA_T83I		
**6**	**9957**	773		NDM, sul-1, rmtB, mut gyrA_T83I		
**7**	**9958**	773		NDM, sul-1, rmtB, mut gyrA-T83I	aadA11, aph[3']-IIb, blaNDM-1, blaOXA-395, blaPDC-16, catB7, crpP, fosA, qacEdelta1, qnrVC1, rmtB4, sul1, tet[G], floR2	parC_S87L gyrA_T83I

**8**	**9951**	773			NDM, sul-1, rmtB, mut gyrA_T83I		
**9**	**9960**	773			NDM, sul-1, rmtB, mut gyrA_T83I		
**10**	**9962**	773			NDM, sul-1, rmtB, mut. gyrA_T83I		
**11**	**9963**	773			NDM, sul-1, rmtB, mut gyrA_T83I		
**12**	**9964**	773	26	46	NDM, sul-1, rmtB, mut gyrA_T83I	aadA11, aph[3']-IIb, blaNDM-1, blaOXA-395, blaPDC-16, catB7, crpP, fosA, qacEdelta1, qnrVC1, rmtB4, sul1, tet[G], floR2	parC_S87L gyrA_T83I
**13**	**9965**	308	23	22	NDM, sul-1, aac[6′]-Ib, qnrS, qnrB, mut gyrA_T83I	aac[3]-Id, aac[6']-Ib, aac[6']-Il, aadA11, aph[3']-IIb, rmtF2, blaNDM-1, blaOXA-10, blaOXA-488, blaPAC-1, blaPDC-19a, ble, catB7, crpP, dfrB5, fosA, msrE, qacEdelta1, qnrVC1, sul1	parC_S87L, gyrA_T83I
**14**	**9966**	773			NDM, sul-1, rmtB, mut gyrA_T83I		
**15**	**9952**	773			NDM, sul-1, rmtB, mut gyrA_T83I		
**16**	**9912**	773	26	46	NDM, sul-1, rmtB, mut gyrA_T83I	aadA11, aph[3']-IIb, blaNDM-1, blaOXA-395, blaPDC-16, catB7, crpP, fosA, qacEdelta1, qnrVC1, rmtB4, sul1, tet[G], floR2	parC_S87L gyrA_T83I
**17**	**10071**	773			NDM, sul-1, rmtB, mut gyrA-T83I	aadA11, aph[3']-IIb, blaNDM-1, blaOXA-395, blaPDC-16, catB7, fosA, qacEdelta1, qnrVC1, rmtB4, sul1, tet[G], floR2	parC_S87L gyrA_T83I

Supplementary Table S4. Fluorometer and Spectrophotometer results of the DNA extraction for NGS by Illumina and Nanopore.

**Illumina**						
**Sample name**	**Source / Species**	**Concentration**	**A260/280**	**A260/230**	**Sample Amount**	**Yield**
9912	Pseudomonas aeruginosa	54 ng/µl	2.01	1.9	100 µl	5.4 µg
9953	Pseudomonas aeruginosa	37.2 ng/µl	1.91	1.22	100 µl	3.72 µg
9958	Pseudomonas aeruginosa	22.5 ng/µl	1.91	2.23	100 µl	2.25 µg
9964	Pseudomonas aeruginosa	18.8 ng/µl	2.02	1.92	100 µl	1.88 µg
9965	Pseudomonas aeruginosa	25.4 ng/µl	1.84	1.1	100 µl	2.54 µg
10071	Pseudomonas aeruginosa	26 ng/µl	2	1.78	100 µl	2.6 µg
Nanopore						
**Sample name**	**Source / Species**	**Concentration**	**A260/280**	**A260/230**	**Sample Amount**	**Yield**
9912	Pseudomonas aeruginosa	33.8 ng/µl	1.90	2.12	100 µl	3.38 µg
9965	Pseudomonas aeruginosa	33.1 ng/µl	1.88	2.14	100 µl	3.31 µg
